# Patch antenna sensor for wireless ice and frost detection

**DOI:** 10.1038/s41598-021-93082-2

**Published:** 2021-07-01

**Authors:** Ryan Kozak, Kasra Khorsand, Telnaz Zarifi, Kevin Golovin, Mohammad H. Zarifi

**Affiliations:** 1 Okanagan Microelectronics and Gigahertz Applications Laboratory, School of Engineering, University of British Columbia, Kelowna, BC V1V 1V7 Canada; 2 Okanagan Polymer Engineering Research and Applications Laboratory, School of Engineering, University of British Columbia, Kelowna, BC V1V 1V7 Canada

**Keywords:** Electronic and spintronic devices, Electrical and electronic engineering

## Abstract

A patch antenna sensor with T-shaped slots operating at 2.378 GHz was developed and investigated for wireless ice and frost detection applications. Detection was performed by monitoring the resonant amplitude and resonant frequency of the transmission coefficient between the antenna sensor and a wide band receiver. This sensor was capable of distinguishing between frost, ice, and water with total shifts in resonant frequency of 32 MHz and 36 MHz in the presence of frost and ice, respectively, when compared to the bare sensor. Additionally, the antenna was sensitive to both ice thickness and the surface area covered in ice displaying resonant frequency shifts of 2 MHz and 8 MHz respectively between 80 and 160 μL of ice. By fitting an exponential function to the recorded data, the freezing rate was also extracted. The analysis within this work distinguishes the antenna sensor as a highly accurate and robust method for wireless ice accretion detection and monitoring. This technology has applications in a variety of industries including the energy sector for detection of ice on wind turbines and power lines.

## Introduction

Ice formation and accumulation creates serious hazards and can cause critical damage or malfunction to equipment in various industries. The impact of icing in the wind turbine power generation industry is particularly concerning due to incidents like the recent winter storm event in Texas where ice accretion stopped the operation of the wind turbines resulting in critical power production loss for multiple days^1^. Additionally, ice accretion reduces the efficiency of wind turbines regularly by altering their aerodynamic profile and disrupts the energy sector by causing power outages when ice accumulates on power transmission lines^2–5^. Ice accretion is also extremely hazardous to the public on roadway infrastructure^6,7^. Moreover, ice accumulation in oil pipelines, if unnoticed, can result in the destruction of the pipeline and environmental contamination^8^. These ice-related hazards exemplify the need for an efficient and accurate system for detecting ice accretion, thickness, and rate of growth.

Various methods have previously been implemented for ice sensing including piezoelectric sensors, ultrasonic-based methods, and guided wave approaches^9–13^. Recently, sensors operating at microwave frequencies have attracted attention due to their small footprint, ease of fabrication, robust nature, and real-time sensing capabilities^14–20^. These advantages have inspired the use of microwave resonators for various sensing applications including ice sensing^21–38^. Microwave resonator sensors operate by monitoring the interaction between high concentrations of electric field (E-field) and a material under test, allowing for material property changes to be observed in real time^39^. In ice detection studies utilizing resonator structures, the microwave sensors were able to detect the accumulation of ice, the thickness of the accreted ice, and the rate of deposition on their surface. However, for real-world implementation on large structures such as wind turbine blades and aircraft wings, transferring the sensed data efficiently can be problematic. In place of the resonator, an antenna sensing structure could overcome this issue by monitoring the *S*_21_ parameters between a transmitting sensing antenna and a receiving antenna, thus enabling a wireless ice detection system.

An antenna can be designed to act as a sensing platform while simultaneously transmitting a signal^40–42^. These structures, known as antenna sensors, have previously been utilized to sense a variety of materials in both liquid and solid form, as well as temperature and crack propagation on surfaces^43–51^. Here, an antenna sensor was developed for wireless ice accretion sensing. A conventional edge fed patch antenna was modified to operate as an antenna sensor. The microstrip patch antenna was advantageous due to its planar design, directive radiation pattern, satisfactory gain, and resonant profile in its transmission and reflection coefficients. Moreover, the localized high concentration of E-field on the edges of this antenna design increases detection sensitivity. The designed antenna sensor was a T-shaped slotted patch antenna with a rectangular slab element along the width of the patch to enhance sensing capabilities.

This paper is organized as follows: The antenna sensor theory of operation and simulations are described in section II, Section III contains the measurement results for the antenna sensor from three consecutive scenarios of a cooling droplet, a frozen droplet, and a thawing droplet, Section IV presents the conclusions of this technique.

### Theory of operation

An edge fed microstrip antenna was designed for this application because a planar structure was ideal for surface ice accretion detection. To achieve small feature size, the antenna sensor was designed on a RT/duroid 6006 substrate which had a relative permittivity of ε_r_ = 6.15 and a thickness of 2.54 mm. The operational frequency of the antenna was designed for 2.45 GHz to take advantage of the available ISM band. Ansys High Frequency Structure Simulator (HFSS) was used for the design and simulation of the antenna sensor. The width, *W*, and the length, *L*, of the patch were derived from Eqs. (1–4)^52^:

1 $${\varepsilon }_{\mathrm{e}\mathrm{f}\mathrm{f}}=\frac{{\varepsilon }_{\mathrm{r}}+1}{2}+\frac{{\varepsilon }_{\mathrm{r}}-1}{2}\left(\frac{1}{\sqrt{1+12\left(\frac{h}{W}\right)}}\right)$$

2 $$W=\frac{c}{2{f}_{0}\sqrt{\frac{{\varepsilon }_{\mathrm{r}}+1}{2}}}$$

3 $$\Delta L=0.412h\left(\frac{({\varepsilon }_{\mathrm{e}\mathrm{f}\mathrm{f}}+0.3)\left(\frac{W}{h}+0.264\right)}{({\varepsilon }_{\mathrm{e}\mathrm{f}\mathrm{f}}-0.258)\left(\frac{W}{h}+0.8\right)}\right)$$

4 $$L=\frac{c}{2{f}_{0}\sqrt{{\varepsilon }_{\mathrm{e}\mathrm{f}\mathrm{f}}}}-2\Delta L$$

**Figure 1:**
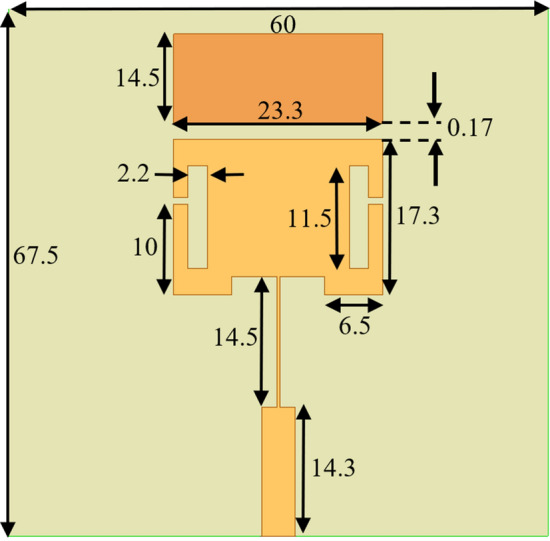
The designed antenna sensor for ice detection. All of the dimensions in this figure are measured in millimeters (mm).

The permittivity and the effective permittivity of the patch are ${\varepsilon }_{\mathrm{r}}$ and ${\varepsilon }_{\mathrm{e}\mathrm{f}\mathrm{f}}$, respectively, f_0_ is the operation frequency of the antenna, and *h* is the thickness of the substrate. Initial calculations based on (1)–(4) demonstrated that, for the designed patch, ${\varepsilon }_{\mathrm{e}\mathrm{f}\mathrm{f}}=5.27$. The antenna was designed on a ground plane with dimensions of 67.5 mm by 60 mm. In order to increase the E-field concentration on the surface of the antenna and reduce the physical dimensions while operating at a lower frequency, two T-shaped slots were added to the patch. A rectangular shaped slab element was also included along the top side of the antenna with the same width to create an area of heightened sensitivity. When a material is placed on the gap between the radiating patch and the rectangular slab, the coupling between the structures increases, shifting the resonant frequency of the antenna sensor. The dimensions of the designed antenna sensor are presented in Fig. 1.

### Modelling and simulation

**Figure 2:**
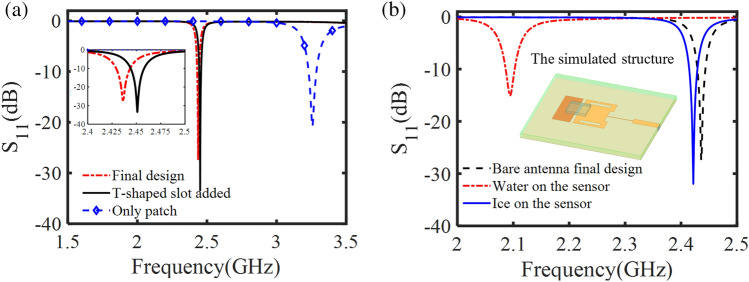
(**a**) Reflection coefficient of simulated antenna for three consecutive design steps. (**b**) Reflection coefficient of simulated antenna for bare, water, and ice scenarios.

Electromagnetic simulations were performed in HFSS over the frequency range of 1.5 to 3.5 GHz using 4001 points. The surface copper of the antenna sensor was modeled using finite conductivity boundary conditions and a radiation boundary condition was used for the far-field simulation of the antenna sensor. The integration of the T-shaped slots with the patch antenna was compared against a conventional patch of the same size. As observed in the result of Fig. 2a, the T-shaped slots shift the operating frequency of the antenna from 3.26 to 2.45 GHz while decreasing the resonant amplitude of the antenna from − 20.81 to − 33.45 dB. Further simulations were performed for the patch antenna sensor with a rectangular slab element placed along the width of the patch. The width and length of the element are 23.3 mm and 14.5 mm respectively. The slab element caused a 14 MHz shift in the resonant frequency of the antenna and a 6 dB shift in resonant amplitude, resulting in a final operational frequency of 2.436 GHz.

To simulate ice detection conditions, water and ice were placed between the patch and the slab element to observe their influence on the antenna sensor’s response. Bridging the gap with water resulted in a 340 MHz shift in the resonant frequency of the antenna sensor, while ice shifted the resonant frequency by 14 MHz (Fig. 2b). The substantial difference in resonant frequency between the two materials is comparable to the difference in permittivity between ice, ${\varepsilon }_{\mathrm{r}}=3.2$, and water, ${\varepsilon }_{\mathrm{r}}=81$.

**Figure 3:**
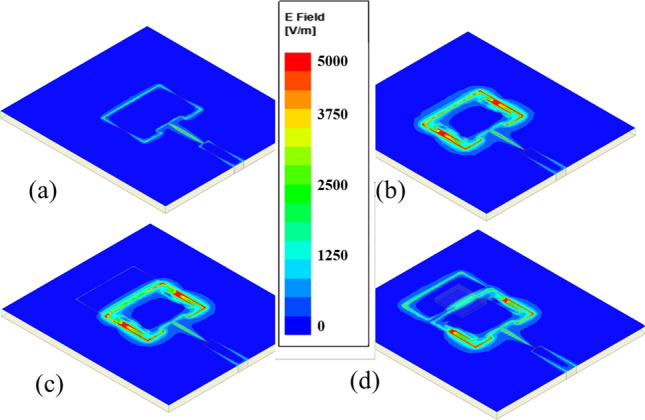
E-field concentration on the surface of the patch antenna (**a**) without modification, (**b**) with T-shaped slots, (**c**) with T-shaped slots and the rectangular slab element, and (**d**) with T-shaped slots, the rectangular slab, and water placed on its surface.

Further simulations were performed to investigate the E-field concentration on the antenna sensor and to understand the effect of the T-shaped slots and the slab element on the antenna’s sensing performance. The antenna sensor exhibited higher E-field concentration on its surface with the T-shaped slots present and the rectangular slab element did not impact the E-field concentration on the bare antenna sensor (Fig. 3). However, when positioning a sample with similar properties of water between the slab element and the patch, the E-field concentration increased on the slab element, indicating a highly sensitive device. The results for the magnetic field (H-field) concentration on the antenna’s surface are provided in Fig. S1. Overall, the observed simulations validate the sensing capability of the device.

**Figure 4:**
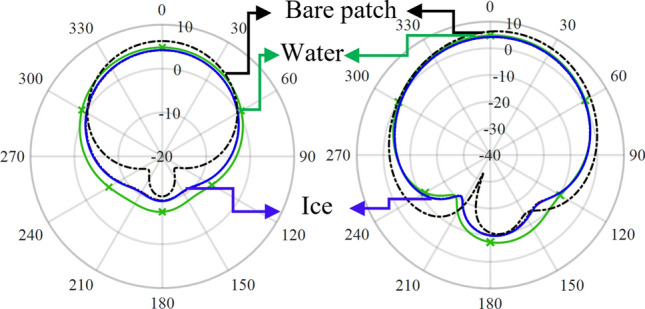
Antenna gain in the (**a**) H-plane and the (**b**) E-plane.

The far-field gain of the antenna sensor was investigated for various simulated conditions in order to validate the sensor’s transmission capability. As observed in the results of Fig. 4, while the bare patch antenna exhibited a gain of 6.3 dB, the T-shaped slots and rectangular slab modifications resulted in a gain of 4.1 dB and 4.2 dB, respectively (Fig. 4). Moreover, placing ice on the gap between the patch and the slab element resulted in a gain of 4.3 dB while the resultant gain was 4.9 dB when water was placed on the same position. The simulations demonstrated that the antenna sensor was capable of detecting and distinguishing between water and ice while simultaneously transmitting the signal to a distant receiver.

**Figure 5:**
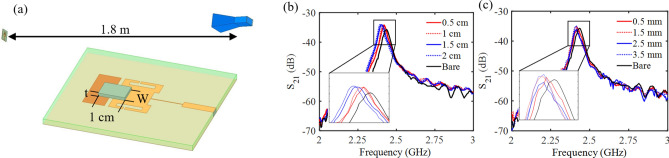
The (**a**) dual-antenna system simulated in HFSS with varying widths and thicknesses of ice on its most sensitive gap and the resonant profile of the transmission coefficient as (**b**) the width of ice varied from 0.5 to 2 cm and as (**c**) the ice thickness varied from 0.5 to 3.5 mm.

To investigate the dual-antenna system for sensitivity to iced area and ice thickness, simulations were performed in HFSS implementing the antenna sensor and a wideband receiving antenna. The results of the simulations for iced area can be seen in Fig. 5b. In this simulation a material with properties similar to ice was placed across the most sensitive gap with a thickness of 1.5 mm and length of 1 cm. The width of the ice was then increased to measure sensitivity to iced area. As the area of the gap covered in ice increased, the resonant frequency of the antenna decreased indicating that the system will be sensitive to iced area. A similar study was also performed on the antenna sensors sensitivity to ice thickness (Fig. 5c). Again, the antenna displayed sensitivity to thickness in the form of down shifting the resonant frequency as the slab of ice increased from 0.5 mm to 3.5 mm with constant width and length of 1.2 cm and 1 cm respectively. These simulations have indicated that the antenna sensor will be capable of detecting both ice thickness and iced area.

## Results and discussion

### Antenna validation

**Figure 6:**
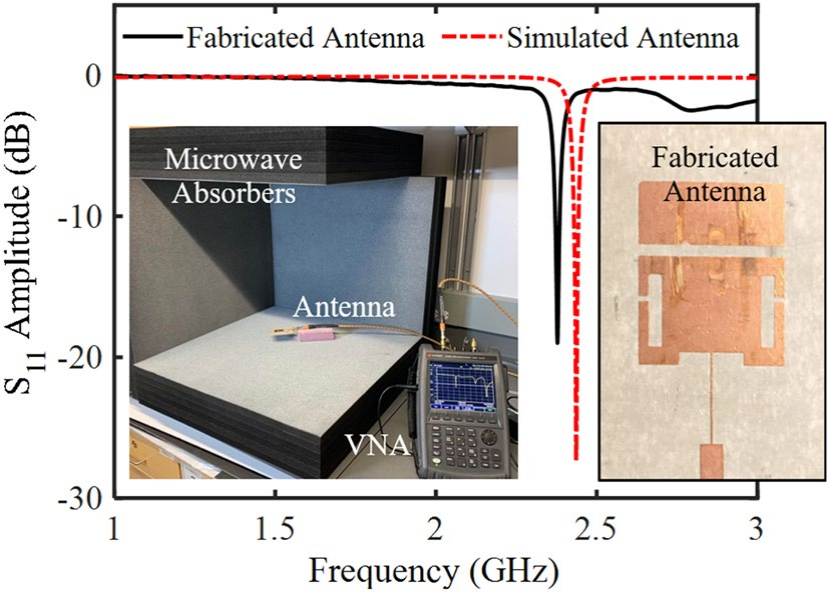
The *S*_11_ response of the simulated and fabricated antenna along with the experimental setup used to make this measurement (left) and the fabricated patch antenna (right). Note that two microwave absorbers have been removed for the photo.

The antenna was fabricated using a chemical etching method and validation of the fabricated antenna structure was conducted by comparing the simulated and measured response. The right inset in Fig. 6 shows the fabricated antenna sensor structure. The performance of the antenna was tested while microwave absorber foams (AN 79) surrounded the antenna. The scattering reflection coefficient (*S*_11_) was measured using a Vector Network Analyzer (VNA) over a frequency range of 1–3 GHz. The operation frequency of the antenna sensor was designed at 2.4–2.5 GHz to exploit the availability of the ISM band. An *S*_11_ comparison between the simulated and measured structure was performed (Fig. 6), and a good match between the simulation and the measurement was observed. The resonant frequency and resonant amplitude differed by 58 MHz and 8.44 dB respectively, for the simulated and measured antenna. These differences were due to the impact of the SMA connectors and soldering, which were not considered in the finite element HFSS analysis. This shift in operation did not impact the performance of the antenna for ice sensing purposes and was therefore considered to be inconsequential. The operational frequency of the fabricated antenna sensor was 2.378 GHz with a resonant amplitude of − 18.98 dB. This frequency lies within the high loss region of water, maximizing the sensitivity of the device for ice detection. The fabricated antenna’s radiation pattern was also measured and compared in Fig. S2. A good match between the simulation results and the experimental results was observed.

### Frost and ice sensing

**Figure 7:**
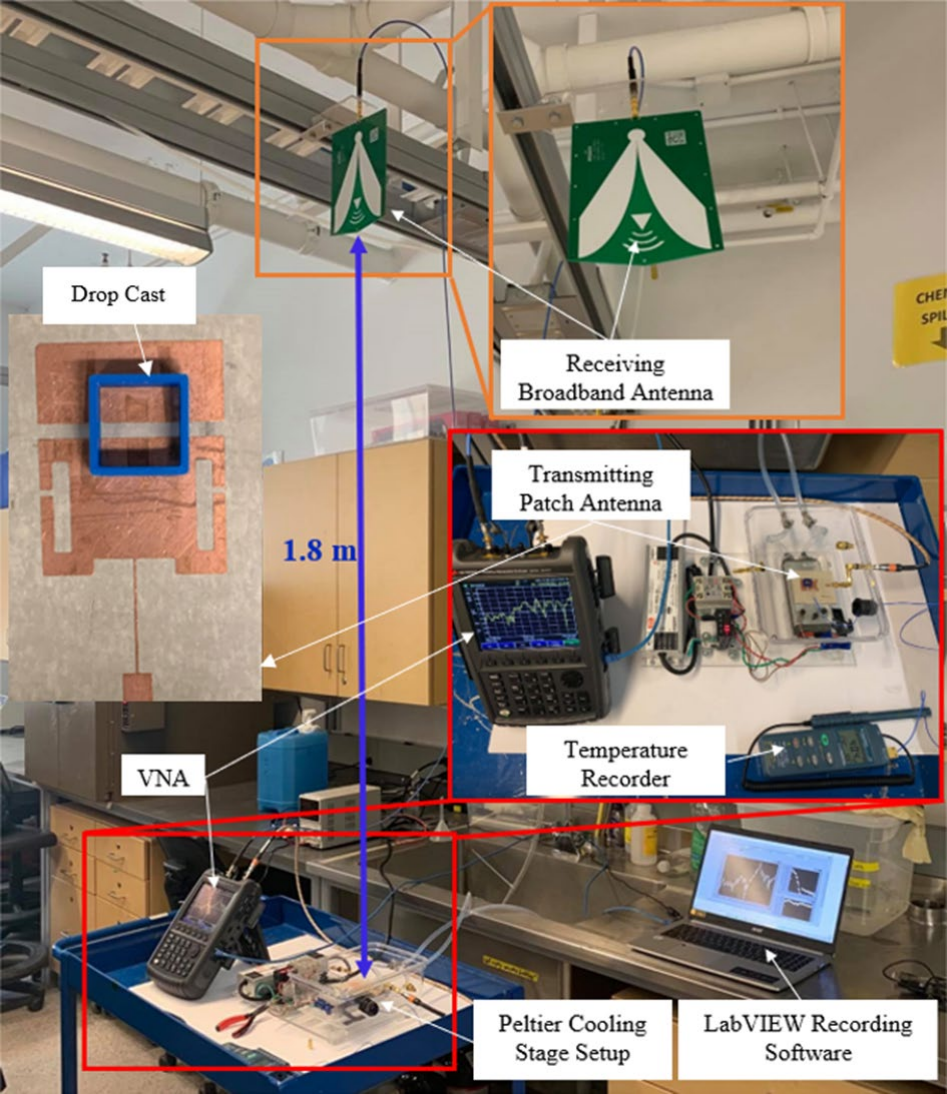
The experimental setup and fabricated patch antenna. The setup utilized a Peltier cooling stage to freeze ice onto the sensing antenna which then transmitted to a receiving antenna positioned 1.8 m above the sensor.

Initial experiments were conducted to determine the sensitivity of the antenna sensor to frost and ice accretion. To perform all of the following experiments, a custom experimental setup was created with the ability to cool the patch antenna below the freezing temperature of water using a Peltier cooling stage. The response of the antenna was monitored using a portable Field Fox N9918A Vector Network Analyzer (VNA) and the *S*_21_ spectrums were recorded over time using a custom LabVIEW program. While recording the results, the VNA was set to sweep 1601 points over a frequency range of 2 to 3 GHz. The bandwidth was set to 1 kHz and the power transmitted was set to 3 dBm. During all experiments, the distance between the transmitting and receiving antennas was constant to avoid changes in the received power. This distance was set at 1.8 m due to experimental space constraints although other distances would work equally well. Coupling of the near fields between antennas was avoided by positioning the receiving antenna many wavelengths away. This results in the received signal consisting only of the far field component radiated from the transmission antenna. The temperature of the antenna sensor was recorded throughout the experiments with a thermocouple temperature probe located near the sensing region. While the custom sensing patch antenna was fabricated for this experiment, a commercial wideband receiving antenna was utilized (RF Space, Model TSA900), with an operational frequency range from 900 MHz to 12 GHz. The experimental setup can be seen in Fig. 7.

**Figure 8:**
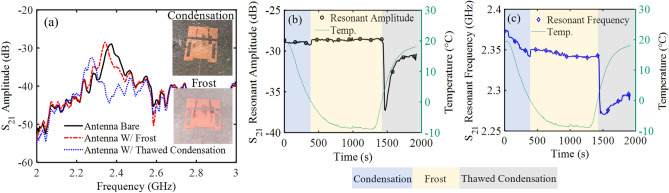
Measured *S*_21_ parameters after allowing condensed water and frost to develop on the antenna. The (**a**) spectrum response of the transmitted signal, (**b**) resonant amplitude tracked over time, and (**c**) resonant frequency tracked over time.

Two validation experiments were conducted to confirm that the antenna could detect water and ice accretion. First, condensation and subsequently frost were formed on the patch antenna by cooling the device to – 9 °C using the Peltier cooling stage. Water vapor from the surrounding environment condensed on the patch antenna and froze, changing the load impedance of the antenna and ultimately shifting its operational frequency and amplitude (Fig. 8).

The transmitted *S*_21_ resonant amplitude and resonant frequency of the bare patch antenna was recorded to be − 28.98 dB at 2.375 GHz at room temperature. As the antenna was cooled and condensed water and frost accumulated on the device, the resonant amplitude and resonant frequency shifted to − 32.61 dB at 2.277 GHz and − 28.49 dB at 2.343 GHz, respectively. The resonant amplitude and frequency both decreased during condensation due to the increased loss caused by water accumulating on the surface. The presence of water changed the load conditions on the antenna, which led to changes in the resonant amplitude and frequency. As the condensation froze into frost approximately 400 s into the experiment, the amplitude sharply increased and continued to gradually increase as more frost developed (Fig. 8b). At the same time, the resonant frequency continued to decrease with the addition of more frost (Fig. 8c). Due to the conductivity and permittivity differences between water and ice, the load on the antenna substantially changed after the water froze, resulting in the observed shifts.

The Peltier stage was turned off around 1300 s to warm the sensor and thaw the developed frost. During thawing, which began around 1400 s, a sharp drop in resonant frequency and resonant amplitude was observed as the frost melted and became liquid water. The increased water on the sensor resulted in high loss followed by a more gradual return to a steady state. This return was caused by the coalescence of the small, melted frost droplets into larger, discrete droplets. The remaining water did not persist as a thin layer over the entire surface, where no increase in amplitude or frequency would be expected. Regardless, these results clearly demonstrate the antenna sensor’s ability to differentiate frost from liquid water.

The total change in resonant amplitude and resonant frequency with respect to the bare antenna due to the presence of frost were 0.49 dB and 0.032 GHz respectively. With condensed water on the antenna the total change in resonant amplitude and resonant frequency were − 3.63 dB and 0.098 GHz respectively. As frost accumulated on the antenna, the resonant amplitude increased 0.49 dB. This was due to the smaller difference in relative permittivity between ice and the substrate, ε_r_ = 3.2 and ε_r_ = 6.15 respectively, compared to the difference in relative permittivity between air and the substrate, improving signal transmission. Contrasting this, the presence of water degraded signal transmission and reduced the resonant amplitude − 3.63 dB. This occurred because water has a much higher relative permittivity of ε_r_ = 81. The resonant frequency will always shift downward in the presence of any additional substance, so long as the material under test has a permittivity higher than air, which increases coupling to the secondary capacitive plate.

**Figure 9:**
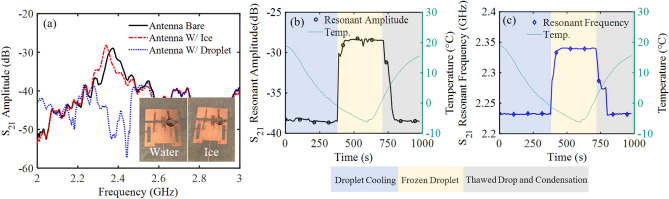
The *S*_21_ response of the antenna while freezing a water droplet to the most sensitive region. The (**a**) spectrum response of the transmitted signal is displayed for the three key regions and the (**b**) resonant amplitude and (**c**) resonant frequency were tracked over time.

The second validation experiment of the antenna sensor involved the freezing of a single droplet of water into ice using the same experimental setup observed in Fig. 7. An 80 µL water droplet was placed on the antenna’s most sensitive region, the gap between the patch antenna and additional capacitive plate, and was frozen while the transient response of the antenna was tracked over time. The purpose of this experiment was to demonstrate the detection with larger volumes of water and ice. The results of this experiment can be observed in Fig. 9.

The resonant amplitude and resonant frequency of the bare antenna, − 28.94 dB at 2.375 GHz, shifted to − 28.24 dB at 2.339 GHz and − 38.29 dB at 2.233 GHz for the single frozen and liquid water droplet, respectively. In the transient graphs, a liquid water droplet was already deposited on the antenna at 0 s. As the antenna was cooled, the droplet froze at 400 s and both the resonant amplitude and resonant frequency increased. This increase in detection parameters due to ice formation was more pronounced compared to the frost experiment due to the increased volume, thickness, and because the ice completely bridged the gap region on the antenna. The signal’s resonant amplitude contained noise in its readout at steady state with ice frozen on the antenna, which was introduced by interference in the environment during detection. This noise indicated that the resonant frequency was more promising to use for stable and robust ice detection, as it would be less affected by interference due to changing environmental conditions. Once the response of the antenna with ice was stable the antenna was allowed to warm up. The ice melted at 700 s and the resonant amplitude and resonant frequency returned to their initial states with minor differences due to additional condensation. This experiment indicated that the antenna can easily detect the presence of ice and water when only a small portion of the antenna is covered.

The frost and single droplet experiments successfully validated the antenna sensor for ice detection applications. To explore the sensor’s capabilities further, the sensitivity of the device to iced area and ice thickness was also investigated.

### Investigation of surface area and thickness detection of ice

**Figure 10:**
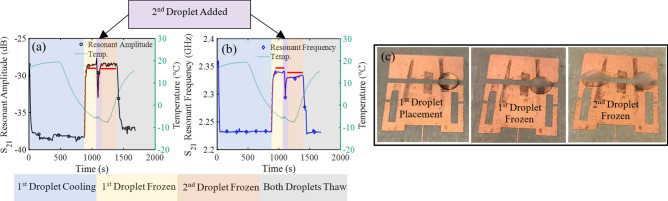
(**a**) Resonant amplitude and (**b**) resonant frequency recorded during the increased iced surface area experiments, and (**c**) the pictured droplet placements on the antenna. Within the plots, the dotted red line is an exponential curve fit and the bold red line is the time averaged data range.

To determine the sensitivity of the antenna sensor to the iced area, an experiment was performed using two water droplets of equal volume. First, an 80 µL water droplet at 18 °C was placed on the antenna and then frozen by reducing the stage temperature to − 5 °C. The sensor’s transient response is presented in Fig. 10. Once the first droplet was frozen and the response of the sensor reached a steady state condition (at 1100 s in Fig. 10), a second 80 µL water droplet was placed on a different area of the sensitive region of the antenna. A significant change was observed in both resonant frequency and the peak amplitude of the antenna sensor. Again, the second droplet froze and the antenna sensor was allowed to reach steady state before both droplets were thawed, at 1400 s, by warming the stage above 0 °C.

Establishing a threshold for ice detection requires the antenna sensor to be capable of detecting changes in the iced surface area coverage. In the above experiment, both the resonant frequency and resonant amplitude shifted as the iced surface area increased. With a single droplet of ice, the time averaged resonant frequency was 2.339 GHz and resonant amplitude was − 28.48 dB. With two droplets of ice the time averaged resonant frequency and resonant amplitude shifted to 2.331 GHz and − 28.50 dB respectively. The time averaged data was used here to eliminate the impact of noise while comparing the response of the sensor. Note that the resonant amplitude experienced a higher level of noise while the resonant frequency remained significantly more stable. As mentioned previously, this was because the resonant amplitude is more susceptible to environmental changes such as movement around the antenna. Although this noise did not preclude the resonant amplitude from being used to threshold detection, the resonant frequency would appear to be a more favorable detection parameter. The total shift in resonant frequency and resonant amplitude due to the increased surface area was 0.023 dB and 8 MHz respectively. The antenna was highly sensitive to the area covered with ice, evidenced by an 8 MHz resonant frequency shift, and can therefore be used to create a threshold of ice coverage before detection is confirmed by other means.

**Figure 11:**
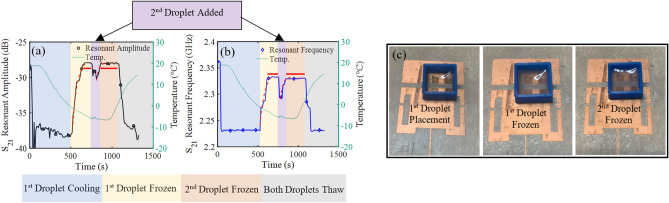
(**a**) Resonant amplitude and (**b**) resonant frequency recorded during the ice thickness experiments, and (**c**) the pictured droplet holder on the antenna with water and ice. Within the plots, the dotted red line is an exponential curve fit and the bold red line is the time averaged data range.

Similarly, a study determining the antenna’s sensitivity to ice thickness was also conducted using two droplets in sequence. This experiment was performed by freezing one 80 µL droplet onto the antenna inside a mold, thereby controlling the surface area covered in ice, and then placing a second 80 µL droplet directly on top of the first. This enabled a study on the impact of ice thickness on the sensor’s transmitted response (Fig. 11). The mold was 3D printed out of PLA thermoplastic and its internal surface area measured 1 cm^2^. To avoid leakage before freezing, the mold was attached to the antenna surface using a small piece of double-sided tape.

The results of this experiment indicated that ice thickness was also detectable through the shift in resonant frequency and resonant amplitude, although the response was not as pronounced. With a controlled surface area covered in ice, the sensors resonant amplitude and resonant frequency were 2.333 GHz and − 27.92 dB after the first droplet froze, and shifted to 2.329 GHz and − 28.05 dB after the second droplet froze. The total change in resonant amplitude was 0.036 dB and the total change in resonant frequency was 2 MHz due to the increased ice thickness. The sensor was less responsive to ice thickness due to the minimal penetration of the near fields into the ice. In general, the EM near field waves will become weaker moving away from the antenna and will minimally penetrate a material. This effect is amplified as the permittivity of the material under test increases. This expectation was evidenced by the minimal change of the sensor’s resonant amplitude and frequency response, 2.25 dB and 39 MHz, respectively, when the second liquid droplet was added. In contrast, for the surface area experiment (Fig. 10), adding the second droplet resulted in a total resonant amplitude shift of 4.49 dB and total resonant frequency shift of 45 MHz. These results indicated that the proximity of the sensor to the ice formation was key for sensitive detection.

For both the ice thickness and iced area experiments, an exponential curve was fit to the transient resonant amplitude and resonant frequency data, for the first and second droplet freezing curves (Table 1). For each curve a time constant, τ, may be extracted that describes the rate at which the water droplet froze into ice. While the values obtained are not overly meaningful, the discrepancies between curves highlight the antenna’s sensitivity to the rate of freezing. For example, during the surface area experiment (Fig. 10) the second droplet froze slightly quicker than the first, due to the antenna being much colder when the second droplet was applied. While this matches the time constants extracted from the resonant frequency data, it does not match the fitted parameters from the resonant amplitude results due to the observed noise. This provides further evidence that resonant frequency is a more robust, sensitive, and accurate measurement parameter for ice detection. Similarly, during the thickness experiment (Fig. 11) the rate of freezing for the second droplet effectively doubled. This was due to the second droplet cooling much faster on the previously frozen ice as well as the initial ice acting as a growth catalyst to begin the formation of the crystal ice structure of the second droplet.

**Table 1:** Curve fitting results on data presented in Figs. 10 and 11 ($Curve\,Fit={y}_{0}+A{e}^{x/\tau })$.

	Resonant amplitude plots			Resonant frequency plots		
	${y}_{0}$	A	τ	${y}_{0}$	A	τ
Surface area experiment (Fig. 10)						
Droplet 1	− 28.9	− 9.35	6.72	2.34E + 9	− 1.08E + 8	10.64
Droplet 2	− 28.6	− 4.27	10.51	2.32E + 9	− 3.56E + 7	8.15
Thickness experiment (Fig. 11)						
Droplet 1	− 27.5	− 9.97	53.22	2.34E + 9	− 1.02E + 8	61.46
Droplet 2	− 27.9	− 2.58	33.22	2.33E + 9	− 4.17E + 7	29.8

According to the presented results, both the resonant frequency and the resonant amplitude of the antenna sensor displayed high sensitivity to the presence of water, ice, and frost over the sensing region. However, the antenna’s resonant frequency response was more reliable because the resonant frequency was not sensitive to the distance between the sensor antenna and the interrogator antenna, as well as being insensitive to possible obstacles between the two antenna that could interfere with the signal. In contrast, the resonant amplitude would be significantly impacted by the distance between the two antennas. The noise observed within all resonant amplitude results originated from interference in the environment. Overall, the resonant frequency is an excellent decision-making parameter when detecting ice as it is not impacted by environmental factors and was practically noise-free within this controlled environment, while the resonant amplitude could be used as supporting data point to confirm the detection.

## Conclusion

In this work, the use of a patch antenna sensor operating at 2.378 GHz to monitor the presence and accumulation of ice was demonstrated. The antenna sensor was determined to be sensitive not only to the presence of ice, but also the thickness, rate of accretion, and surface area covered. It was determined that both the resonant amplitude and resonant frequency of the *S*_21_ transmission between the antenna sensor and a wide band receiver could be used to detect ice accretion. However, due to the resonant amplitude being impacted by environmental factors such as distance to the receiver and obstructions in the line-of-sight path, the resonant frequency was the superior decision-making parameter. This proposed ice sensing method is low-cost, real-time, robust, and highly accurate with the additional advantage of wireless readout capability, eliminating the need for long transmission lines on large structures. The detection of ice in both the public and private sectors will create safer environments and protect equipment from being damaged or destroyed. Further development of this sensor will produce an ice detection technique which can be easily implemented into existing structures such as wind turbines for real-time ice sensing.

## Supplementary Information

Supplementary Information.
