# Supplementary material for: Patch antenna sensor for wireless ice and frost detection
Source: Sci Rep. 2021 Jul 1;11:13707. doi: 10.1038/s41598-021-93082-2 (PMC8249444; doi:10.1038/s41598-021-93082-2)
Supplement: Supplementary file 1 — Supplementary Information. [file 41598_2021_93082_MOESM1_ESM.pdf]

Supplementary

Patch Antenna Sensor for Wireless Ice and Frost Detection

Ryan Kozak[1,3], Kasra Khorsand[1,3], Telnaz Zarifi[2], Kevin Golovin[2] and Mohammad H. Zarifi[1]*

1 Okanagan Microelectronics & Gigahertz Applications Laboratory, School of Engineering, University of British Columbia, Kelowna, BC V1V 1V7, Canada

2 Okanagan Polymer Engineering Research & Applications Laboratory, School of Engineering, University of British Columbia, Kelowna, BC, V1V 1V7, Canada

3 Authors with equal contributions

***Corresponding Author: Mohammad H. Zarifi** Mohammad.zarifi@ubc.ca*

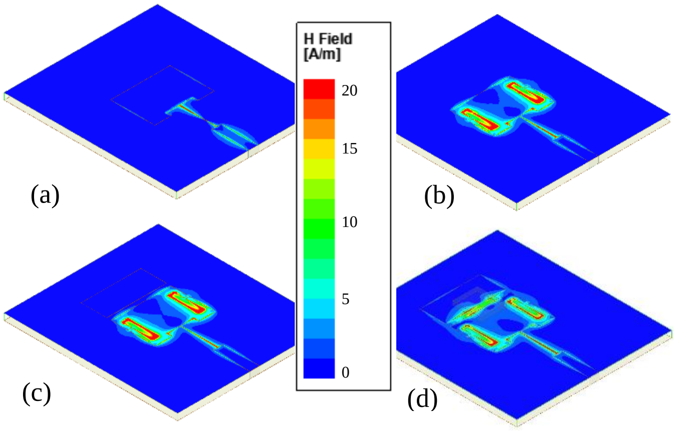

S1. H-field concentration on the surface of (a) patch antenna without any modification. (b) Patch antenna with T-shaped slots. (c) Patch antenna with T-shaped slots and rectangular slab element. (d) Patch antenna with T-shaped slots and rectangular slab while a water sample is placed on its surface.

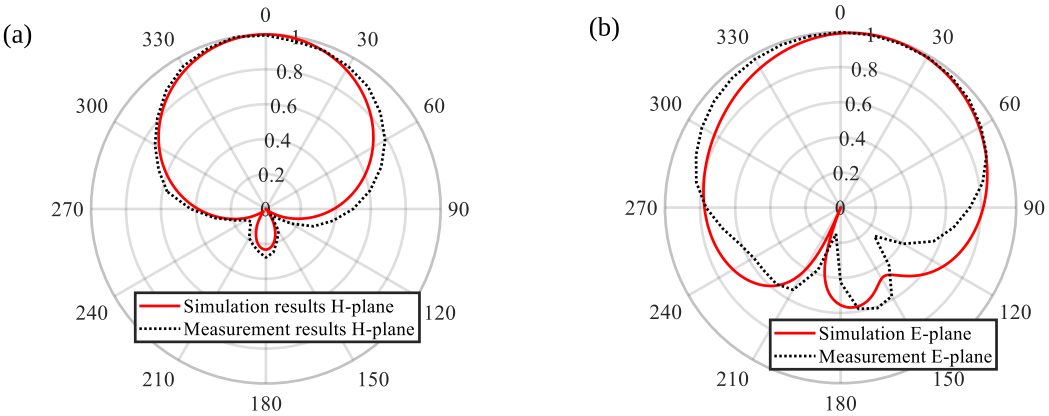

Fig. S. 2: The measured and simulated radiation pattern of the designed patch antenna sensor in the (a) H-plane and (b) E-plane.
